# Evaluating the Usability of an mHealth App for Empowering Cancer Survivors With Disabilities: Heuristic Evaluation and Usability Testing

**DOI:** 10.2196/51522

**Published:** 2024-04-02

**Authors:** Rachel F Adler, Kevin Baez, Paulina Morales, Jocelyn Sotelo, David Victorson, Susan Magasi

**Affiliations:** 1 Department of Computer Science Northeastern Illinois University Chicago, IL United States; 2 School of Information Sciences University of Illinois Urbana-Champaign Champaign, IL United States; 3 Department of Medical Social Sciences Northwestern University Evanston, IL United States; 4 Department of Occupational Therapy University of Illinois Chicago Chicago, IL United States

**Keywords:** mobile health, mHealth, apps, usability, cancer survivors, accessibility, disabilities, cancer, oncology, heuristics, empowerment, advocacy, mindfulness, problem-solving

## Abstract

**Background:**

More than 18 million cancer survivors are living in the United States. The effects of cancer and its treatments can have cognitive, psychological, physical, and social consequences that many survivors find incredibly disabling. Posttreatment support is often unavailable or underused, especially for survivors living with disabilities. This leaves them to deal with new obstacles and struggles on their own, oftentimes feeling lost during this transition. Mobile health (mHealth) interventions have been shown to effectively aid cancer survivors in dealing with many of the aftereffects of cancer and its treatments; these interventions hold immense potential for survivors living with disabilities. We developed a prototype for WeCanManage, an mHealth-delivered self-management intervention to empower cancer survivors living with disabilities through problem-solving, mindfulness, and self-advocacy training.

**Objective:**

Our study conducted a heuristic evaluation of the WeCanManage high-fidelity prototype and assessed its usability among cancer survivors with known disabilities.

**Methods:**

We evaluated the prototype using Nielsen’s 10 principles of heuristic evaluation with 22 human-computer interaction university students. On the basis of the heuristic evaluation findings, we modified the prototype and conducted usability testing on 10 cancer survivors with a variety of known disabilities, examining effectiveness, efficiency, usability, and satisfaction, including a completion of the modified System Usability Scale (SUS).

**Results:**

The findings from the heuristic evaluation were mostly favorable, highlighting the need for a help guide, addressing accessibility concerns, and enhancing the navigation experience. After usability testing, the average SUS score was 81, indicating a good-excellent design. The participants in the usability testing sample expressed positive reactions toward the app’s design, educational content and videos, and the available means of connecting with others. They identified areas for improvement, such as improving accessibility, simplifying navigation within the community forums, and providing a more convenient method to access the help guide.

**Conclusions:**

Overall, usability testing showed positive results for the design of WeCanManage. The course content and features helped participants feel heard, understood, and less alone.

## Introduction

### Background

There are an estimated 18.1 million cancer survivors in the United States, and the number is projected to increase to 22.5 million by 2032 [1]. Approximately 40% of cancer survivors experience long-term physical, cognitive, psychological, and social consequences of cancer and its treatment, which can lead to significant disability [2]. These effects can include physical challenges, including but not limited to pain, fatigue, decreased functional mobility, limb loss, lymphedema, speech and swallowing difficulties, emotional challenges (as cancer survivors may experience anxiety or depression), and cognitive challenges (such as “chemo brain”) [3-5]. These aftereffects can lead to activity limitations and participation restrictions, which according to contemporary frameworks and legal definitions may be considered as disabilities [6,7]. Yet, even with significant functional impairments, not all cancer survivors self-identify as disabled [8,9]. Regardless of the terminology used, the aftereffects of cancer and their related functional impacts can have a significant negative impact on well-being and health-related quality of life [10]. Survivorship plans and rehabilitation programs, which play a crucial role in restoring survivors’ physical and emotional well-being, are frequently underused by cancer survivors [11]. This can be due to obstacles like time, financial constraints, and transportation issues [12], which hinder their accessibility. Mobile health (mHealth) apps can help make rehabilitation services accessible and put them in the hands of those who need them.

### mHealth Apps

Mobile technologies—smartphones, tablets, and smartwatches—are increasingly ubiquitous in today’s society and can be used almost anywhere [13]. The Pew Research Center reports that 85% of American adults own smartphones, and the ownership is relatively consistent across genders; racial groups; and urban, suburban, and rural users [14]. This leads to an increase in the development of mHealth apps. The COVID-19 pandemic has led to mHealth strategies becoming even more important in cancer care. According to the recommendations of Curigliano et al [15], patients with cancer should be offered mHealth strategies to support symptom management and adoption of healthy behaviors. The number of mHealth apps has increased throughout the years, with around 325,000 apps available in 2017 [16]. Charbonneau et al [17] identified 123 mHealth apps for cancer survivors available in the 2 most important marketplaces (ie, Apple iTunes and Google Play). Typical areas of usage in cancer are disease management support (eg, symptom monitoring, management of side effects, medication reminder and dosing, and access to health information), support of healthy behavior (eg, healthy diet and increased physical activity), or the connection with other patients (eg, social support through peers) [18-20].

### Evaluating the Usability of mHealth Apps

It is important to gather qualitative and quantitative data on mHealth apps to determine how satisfied users would be with the product at hand. According to one scoping review, of 133 different eHealth articles that conducted usability testing, 105 used questionnaires, 57 used task completion, 45 used “think aloud,” 37 conducted interviews, 18 performed heuristic evaluation, and 13 used focus groups [21]. The System Usability Scale (SUS) was the most frequently used questionnaire with a total of 44 studies. A combination of methods was used in 88 of the studies. Further, cancer was tied as the second most frequently evaluated health condition (n=10), with only mental health being evaluated more often (n=12).

Usability testing is a common effective method for evaluating the usability of mHealth apps. Studies have shown that usability testing is an effective method for examining mHealth apps for diabetes [22,23], depression [22,24], and youth at risk for developing psychosis [25], as well as managing pain [26], heart failure [27], and cancer symptoms [28]. Common questionnaires often included variations on the Mobile Application Rating Scale [25,27] or the SUS [22,24,26]. Additional techniques often employed in usability testing include measuring time per task [26] and using think aloud techniques [29]. In addition to evaluating fully implemented mobile apps, studies have conducted usability testing on prototypes of mHealth apps for supporting mental health [30], chronic kidney disease [29], fall risk detection system for older users [31], HIV [32], and cancer survivors [33-35]. Many studies have conducted heuristic evaluation before usability testing on an mHealth prototype to fix usability issues before bringing it to users [28,29,32,33]. While Nielsen’s 10-point usability heuristics [36] are geared toward computer-based applications, most of these are also applicable in mobile app design. The SUS questionnaire was also commonly used in usability testing studies for examining mHealth prototypes [29,31,37].

### WeCanManage App

We designed a high-fidelity prototype for WeCanManage, an evidence-informed mHealth self-management intervention, aimed at empowering individuals with tools to effectively manage cancer as a chronic condition. Users are asked to log into the app daily for 5-10 minutes to complete mobile microlearning modules of self-management content. The intervention content is based on extensive literature review and formative interviews with cancer survivors with known disabilities (n=30) and supportive cancer care professionals including social workers, psychologists, occupational and physical therapists, and a physiatrist specializing in cancer rehabilitation (n=5) [9]. A team of survivor scientists, people with lived experiences of cancer and disability, further informed intervention content and focus. Intervention content is presented sequentially as information is scaffolded on itself to promote depth of learning, retention, and application. The content is divided into 4 broad sections: WeCanRelate (fosters a sense of validating and normalizing the survivorship experience), WeCanAdapt (teaches goal direction self-management strategies), WeCanBe (emphasizes mindfulness-based practices), and WeCanSpeakUp (addresses self-advocacy and disability rights). In addition to the instructional content, WeCanManage provides users with 3 circles of support, including one-on-one connections with other users (Connect to Peers [C2P]), community forums (to discuss intervention content and shared experiences with the entire user community), and a library with evidence-informed educational content [38]. We conducted a thorough evaluation of the usability of the high-fidelity prototype for cancer survivors with disabilities, employing both heuristic evaluation and usability testing to assess its effectiveness in addressing the unique needs and challenges of this user group.

## Methods

### WeCanManage High-Fidelity Prototype

**Figure 1:**
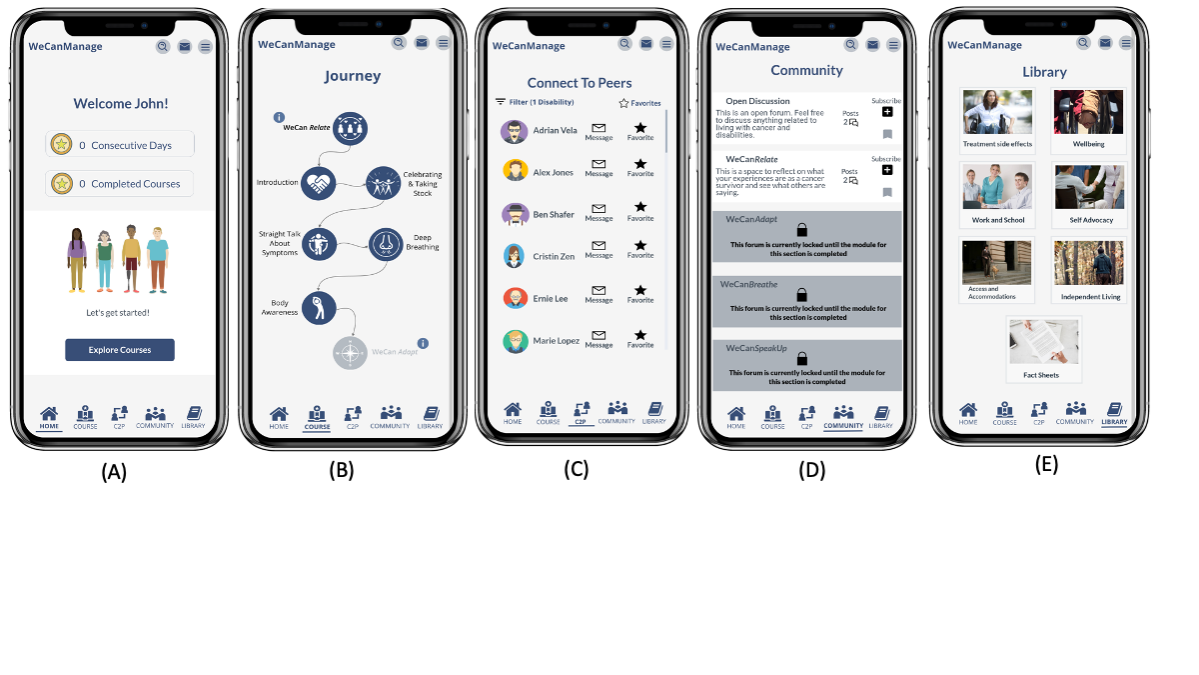
Screenshots of the WeCanManage prototype: (A) Home, (B) Journey, (C) Connect to Peers (C2P), (D) Community, and (E) Library.

The high-fidelity prototype was created on Marvel [39], a web-based collaborative design platform that provides tools for creating wireframes, designs, and prototypes of interactive applications. We aimed to design WeCanManage specifically for smartphone usage. The prototype of WeCanManage allows users to navigate between the Home, Journey (Courses), C2P, Community (Community Forum), and Library (see Figure 1).

**Figure 2:**
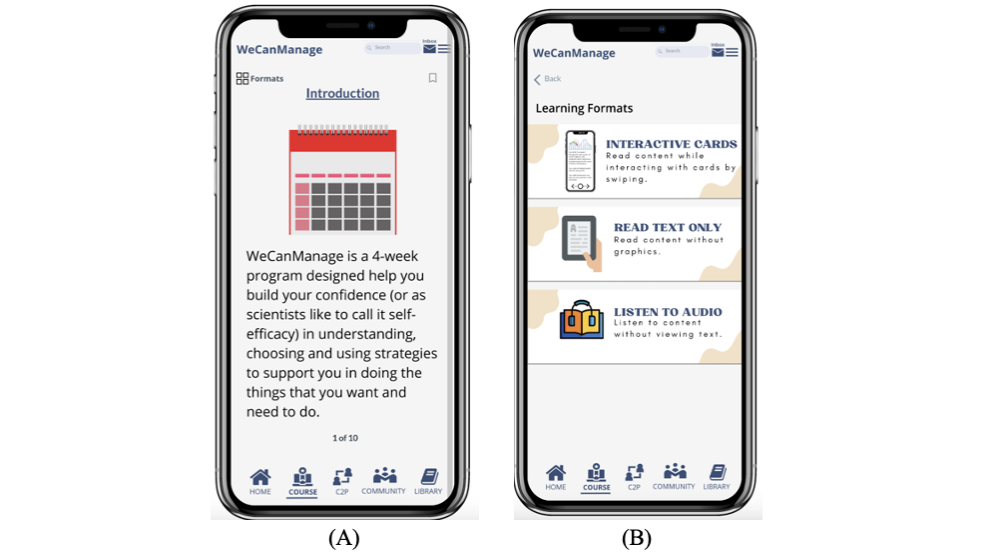
Screenshots before heuristic evaluation: (A) card view and (B) learning format after clicking on the Formats icon.

The Course section provides cancer survivors with an educational intervention that works with them on dealing with the long-term effects of their newly acquired disabilities through problem-solving, mindfulness, and self-advocacy. The content is designed to be a 4-week program where the user unlocks a series of microlessons divided into 4 modules (WeCanRelate, WeCanAdapt, WeCanBreathe, and WeCanSpeakUp), which educate users with different methods to deal with the effects of postcancer treatment in their daily life. To prioritize user control and accessibility, the course content is conveyed through mobile microlearning modules, presented in different formats such as readable text, clickable text-based cards, and audio (Figure 2).

**Figure 3:**
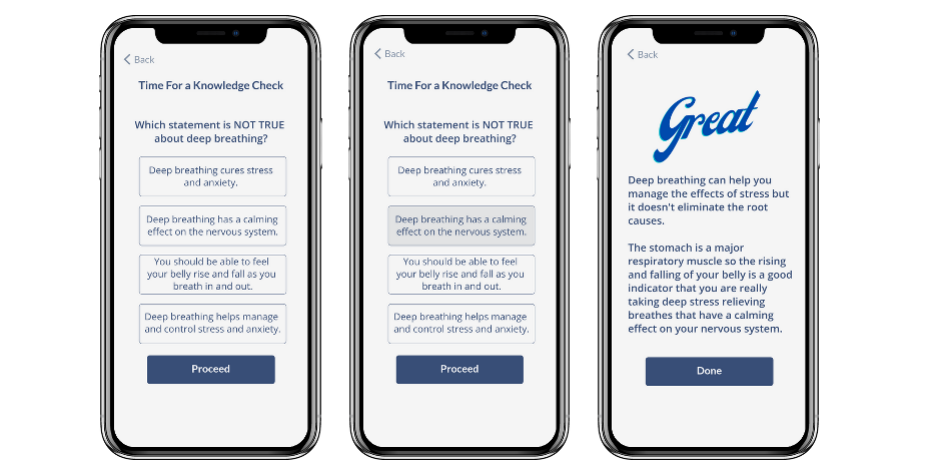
Sample of a knowledge check.

At the end of many of the daily sessions, there are interactive engagement activities, such as reflections that feed into the Community Forum and knowledge checks (see Figure 3). The engagement activities are designed to support consolidation of knowledge and application of course content to the user’s lived experiences.

The Community and C2P sections offer users a chance to engage with others, fostering networking opportunities and creating a support system with individuals undergoing similar experiences. C2P facilitates connections with others, allowing users to filter by categories like cancer type and disability, while Community features discussion forums for each of the 4 course sections and an open discussion forum. Lastly, the Library section contains additional evidence-informed resources such as articles and factsheets. The various sections of the prototypes were initially created as a low-fidelity prototype through an iterative co-design approach involving both the design teams and cancer survivors, who served as representatives of our targeted audience [40].

Because of its prototype nature, users could navigate all links, but functionalities such as real-time chat with other users and composing reflections or community posts were not operational. To overcome this, we incorporated simulated features in the prototype, triggering them automatically on user interaction. After creating the high-fidelity prototype, we evaluated it through 2 distinct methods: heuristic evaluation and usability testing.

### Methodology for Heuristic Evaluation

Nielsen’s 10 principles of heuristic evaluation [36] were used for the initial testing of the prototype (Textbox 1). The prototype was given to 22 undergraduate students at a Midwestern university taking a human-computer interaction course in the Spring of 2022 who were trained in conducting heuristic evaluation. No supplemental demographic data were gathered. They were given the WeCanManage prototype during a class period of 1 hour 15 minutes. During the session, students were split into 6 groups, and each group was given 5 tasks to complete using the prototype. We created 3 sets of 5 tasks, and therefore every 2 groups completed the same tasks. The tasks included going through the introduction course module, switching to text and video fields, and filtering the users by a specific disability through the C2P page. Students logged in to classroom computers and accessed Maze, an online testing platform used to monitor assessment details [41], recorded the path taken by students to complete tasks, and presented questions about their experience to help track their progress. At the end of the session, the groups documented violations of the 10 heuristic principles and rated their usability severity on a 0-4 scale, where 0 is not a usability problem and 4 is a usability catastrophe. Furthermore, the student evaluators filled out a questionnaire through Maze providing feedback and thoughts on the prototype’s design. The questionnaire covered their likes and dislikes of the design, their impressions of course modules, and the ease of changing the format of the content.

**Ten principles of heuristic evaluation from Nielsen [36].**

1. Visibility of system status
2. Match between system and the real world
3. User control and freedom
4. Consistency and standards
5. Error prevention
6. Recognition rather than recall
7. Flexibility and efficiency of use
8. Aesthetic and minimalist design
9. Help users recognize, diagnose, and recover from errors
10. Help and documentation

### Methodology for Usability Testing

We modified the prototype based on the feedback from heuristic evaluation and conducted usability testing over Zoom. We used purposive sampling with targeted outreach through cancer survivorship networks, including both clinical and community. To be eligible for participation, individuals had to meet the following inclusion criteria: be 18 years or older; have a history of breast cancer, head and neck cancer, or sarcoma; have completed active treatment; self-identify as a person with a disability; and possess the ability to understand and communicate in English. Participants received a gift card for their time. Sessions lasted approximately 90 minutes. Sessions were recorded and participants shared their screens for data collection. Participants were told to connect to Zoom on a computer or laptop device. Usability testing occurred between September 2022 and February 2023. As we encountered minor issues with the Maze platform during the heuristic evaluation, including audio malfunctions, we transitioned to Ballpark, an extension of Marvel that facilitated usability testing of the prototype. Participants were given 8 tasks to complete (see Textbox 2). They were told that they were on day 6 of the 4-week period. Consequently, they could access content from sessions 1-6, while subsequent sessions remained locked to replicate the user’s sequential navigation experience, with new content being unlocked on a daily basis. The first 6 tasks were based on the course sessions and navigating through each course by reading the content cards and doing related engagement activities. Task 2 required participants to switch the viewing mode using the accessibility features (eye symbol) to the text-only mode, while task 6 involved watching a 1 minute 20 second–long mindfulness video, instead of the default card format. The final 2 tasks (tasks 7 and 8) focused on navigating the Community Forum and C2P sections. After each task, participants rated their satisfaction level and the time taken to complete each task using a 7-point Likert scale. On finishing all 8 tasks, participants had the opportunity to freely explore the app using a “think aloud” approach to express their thoughts and experiences.

To evaluate usability, participants completed the modified SUS, a reliable and valid 10-item questionnaire that assesses usability [42,43]. While the SUS has been around since 1986, it has been shown to be effective in evaluating the usability of recent health apps [44]. To calculate SUS scores, 1 is subtracted from the raw score of the odd-numbered items (those items phrased in a positive way), and the raw score of the even-numbered items (those items phrased in a negative way) is subtracted from 5. The total scores are then multiplied by 2.5 to derive the “standardized SUS score,” which ranges from 0 to 100. A SUS score of 68 is considered average usability [45], while a score above 80.3 is deemed an A grade, placing it in the top 10% of scores [46] and corresponding to a narrative rating of good-excellent [47]. In addition, we included open-ended questions to gather feedback on participants’ preferences and areas for improvement regarding the app. Examples of these questions include “How easy or difficult was it to see all the content on the screen?” and “What did you think of the design of the course modules?”

To assess the effectiveness of the app design, following a similar approach to Adler et al [48], we evaluated task completion by having 2 independent coders review each recording and code whether the participants

- Completed the task quickly on their own (C)
- Completed the task on their own though it took a little longer (L)
- Needed help to complete the task (H)

The coders achieved an agreement percentage of 87.5%. Any discrepancies were resolved through discussion. To assess efficiency, we analyzed the number of misclicks (clicks outside of clickable areas in the prototype) and the time taken to complete each task.

**Eight tasks given to usability testing participants.**

**Course**

1. Go to the Course and click on the WeCanRelate session. Read through all of the cards.
2. Go to the Course and click on the Introduction session. Switch to Text view to read all the cards at once using the eye symbol on the bottom left of the first screen of the module.
3. Go to the Course and click on the Celebrating & Taking Stock session. Read through all the cards and then go to the reflection. Start “typing” your reflection and post it. Do you see your post accurately reflected?
4. Go to the Course and click on the Straight Talk About Symptoms session. Read through the cards and follow the link to the library and the Understanding the Cancer Rehabilitation Team Fact Sheet.
5. Go to the Course and click on the Deep Breathing session. Read through the content and complete the knowledge check. Did you get the correct answer?
6. Go to the Course and click on the Body Awareness session and go through to the end of the module by watching the video.

**Community**

1. Go to the Community Forum. Create a new post in the Open Discussion forum. Enter a title, select the community tag, enter text, and post your response.

**Connect to Peers**

1. Find the Connect to Peers (C2P) option and filter to narrow the search to people who are deaf or hard of hearing.

### Ethics Approval

We obtained institutional review board approval from the participating universities in the project (University of Illinois Chicago #2020-1067, Northeastern Illinois University #79, and Northwestern University #NUUIC21CC03). 

## Results

### Results of Heuristic Evaluation

**Figure 4:**
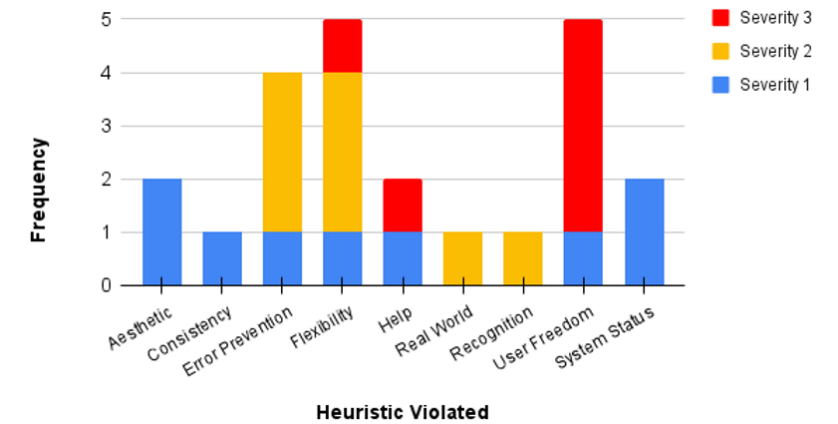
Graph displaying the frequency and severity of heuristic violations.

We conducted an analysis of the identified heuristic violations and their severity. The highest severity rating recorded was a 3, as illustrated in Figure 4. The most frequent heuristic violations were related to flexibility, user control, and freedom, followed by error prevention. The issues identified were primarily navigation problems within the prototype, missing back buttons, and font size being too small. Suggestions for improvement were also raised, such as adding an FAQ page, a way to contact the creators or administrators, and including a walk-through or how-to page. Student evaluators expressed appreciation for the images and content, the knowledge check feature, the color scheme, and the layout. They found the app easy to read and navigate. The dislikes expressed included the absence of a help guide and nonfunctional back buttons. Additionally, some groups reported having difficulty finding the format button to switch the mode of learning to text-only or audio.

### Modifications Based on Heuristic Evaluation

**Figure 5:**
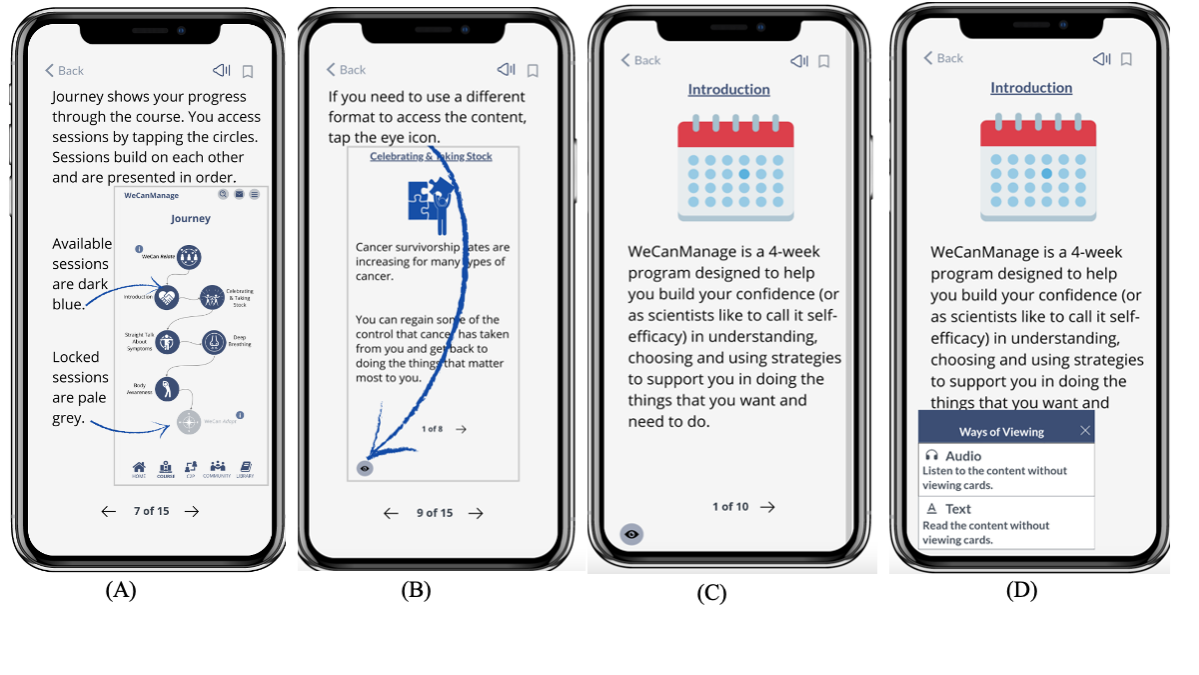
Updated prototype screens after heuristic evaluation. (A,B) Help guide incorporated into the first course session. (C,D) Updates to the accessibility format and switching from card view to audio or text views.

Drawing from the findings of the heuristic evaluation, we enhanced the prototype by introducing a help guide (Figure 5A and B) and seamlessly integrating it into the first course session. We also revised the method for switching accessibility format features (Figure 5C and D). Furthermore, we increased the font size on multiple screens and improved navigation by implementing additional back buttons for a smoother user experience.

### Results of Usability Testing

We had 10 cancer survivors with disabilities (9 female, 1 male; 9 White or Caucasian, 1 Black or African American) who completed usability testing. The average age of the participants was 59 years. Usability scores show that participants had an overall positive reception to the design of the prototype. We had an average SUS score of 81; our prototype’s usability is therefore considered good to excellent with a grade of an A and in the top 10%.

We assessed participants’ satisfaction levels and the time taken to complete each task. The average scores for these 2 measurements are presented in Table 1. Generally, participants exhibited high satisfaction rates; however, lower numbers were observed for task 2 (finding the eye icon to change the accessibility format), task 7 (creating a post in the Community Forum), and task 8 (using the filter in C2P).

**Figure 6:**
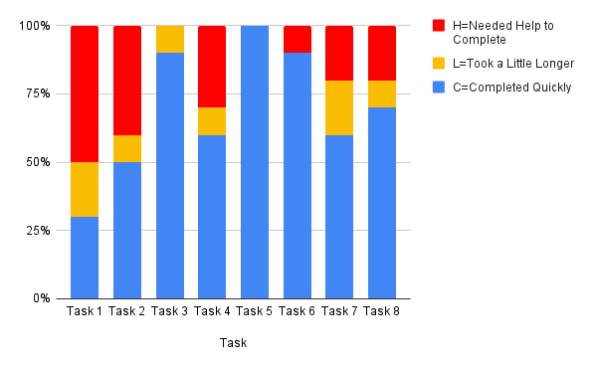
Graph displaying the frequency of H (required assistance to complete the task), C (completed quickly), and L (completed with a little more time) ratings given to participants as they completed a task.

In addition, we evaluated the effectiveness of the app design by categorizing participants’ task completion into 3 groups: completed quickly (C), completed with a little more time (L), or required assistance to complete the task (H). Overall, most participants completed their tasks without any issues, with only 17 of 80 cases (21%) needing help to complete them (see Figure 6). During task 1, a slight learning curve was observed as some participants had difficulty locating the correct module, leading to the need for assistance in completing the task. However, this issue was not prevalent in subsequent tasks. Task 2 revealed that some participants encountered challenges while switching the card format to text view using the eye symbol, as they had trouble locating the button. In task 4, some participants faced difficulties clicking on the correct resource within the Library as directed in the learning module. For tasks 7 and 8, several participants struggled to navigate both the Community and C2P sections because certain text and icons were too small or unclear in their function, leading to confusion on what to do.

Likewise, while analyzing efficiency based on the number of misclicks per task, tasks 7 and 8 exhibited notably higher misclick rates (Table 2). The table also presents the actual time taken per task, with task 1 showing higher time than the other tasks. As mentioned earlier, task 1 had a learning curve, but it also involved reading the most cards (15 cards) as we integrated the help guide into the first course session. Therefore, this finding is expected given the additional content to review in task 1.

The prototype’s help guide received a positive response, with 8 of 10 participants (80%) rating it as very helpful or extremely helpful. Similarly, 8 of 10 participants (80%) reported finding the eye symbol (to change the course format) easily. In response to open-ended questions, participants expressed their likes and dislikes of the prototype and its design. Many participants shared positive opinions on the design and content of the modules, finding them helpful and insightful. The video located within one of the modules received positive feedback, with some expressing a desire for additional videos. The purpose of the Community section was well liked as participants enjoyed having a place to freely express themselves with other cancer survivors and appreciated the opportunity for users to support each other. The Library resources were found to be informative and useful, covering a wide range of topics.

Our findings were overwhelmingly positive, supported by quotes from participants (some written and some oral):

I want to see the whole thing work! I know that this is a prototype, but I want to see more!

Great app, it would have been very helpful to me when I was just out [of] treatment.

Even though I'm not very comfortable with technology, and that might be because of my age, … I don't think that this would be difficult for me. I think there'd be a real fast learning curve. I felt good and positive when I realized I had learned something, and I could just click on it now without having to think about it.

I do like the app. I like that I know I’m not alone feeling this way.

These participant quotes reflect their enthusiasm and positive experiences with the app, highlighting its potential benefits and ease of use.

On the basis of our session observations and participants’ feedback on areas for improvement, we identified several issues:

- Accessibility concerns, including small font sizes and icons, particularly with the navigation arrows on cards, the top navigation bar, and the eye icon.
- Some participants experienced confusion while navigating the Community page when creating new posts.
- Difficulty in locating and using the filter option within the C2P page.
- Participants expressed a desire for an easy way to return to the help guide.
- Feedback indicated a preference for changing the robotic voices used in the audio format for the modules. The prototype used Google US English from voicegenerator.io, but the intention is to have a real person’s voice in future implementations.

Addressing these areas for improvement can further enhance the app’s usability and user experience.

**Table 1:** Average satisfaction per task and time per task (out of 7).

Task	Average task satisfaction	Average time satisfaction
1	6.5	6.4
2	5.7	5.9
3	6.6	6.5
4	6.5	6.2
5	6.7	6.3
6	6.8	6.6
7	5.2	5.5
8	5.8	5.7

**Table 2:** Percentage of misclicks and time per task.

Task	Misclicks (%)	Time (minutes)
1	8	3:28
2	4.75	2:28
3	3.30	2:19
4	5.64	2:13
5	0.83	2:15
6	0	1:57
7	19.24	1:34
8	16.38	0:44

### Modifications Based on Usability Testing

**Figure 7:**
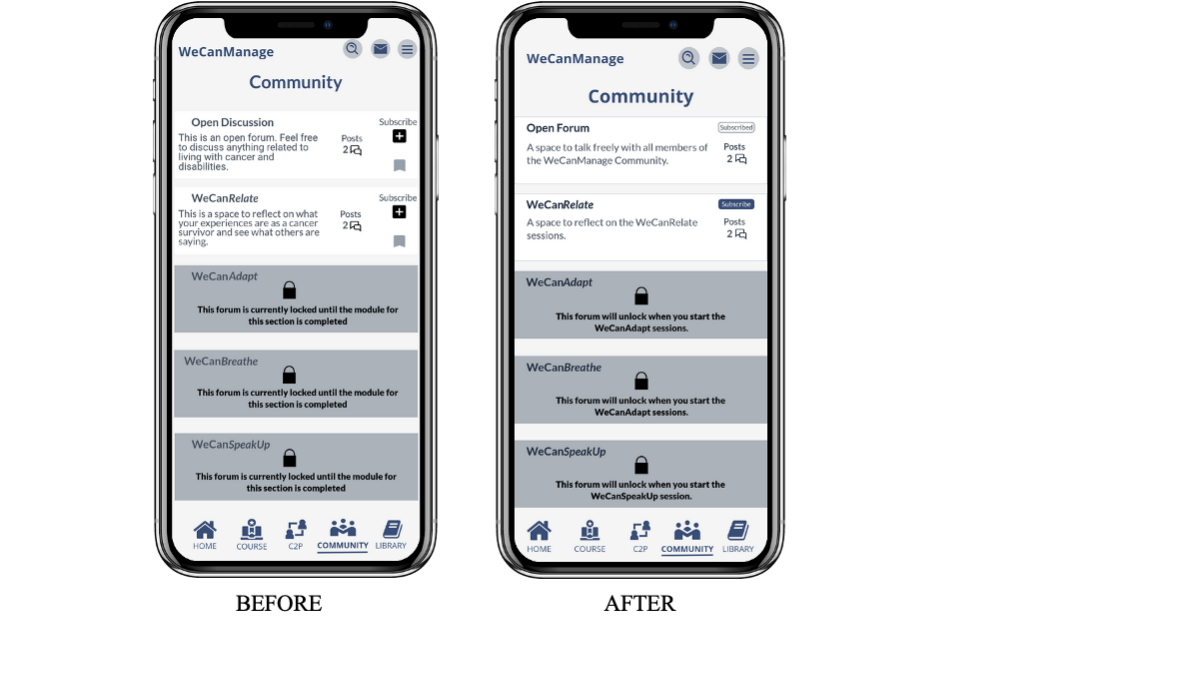
Modifications made to the Community before and after usability testing.

**Figure 8:**
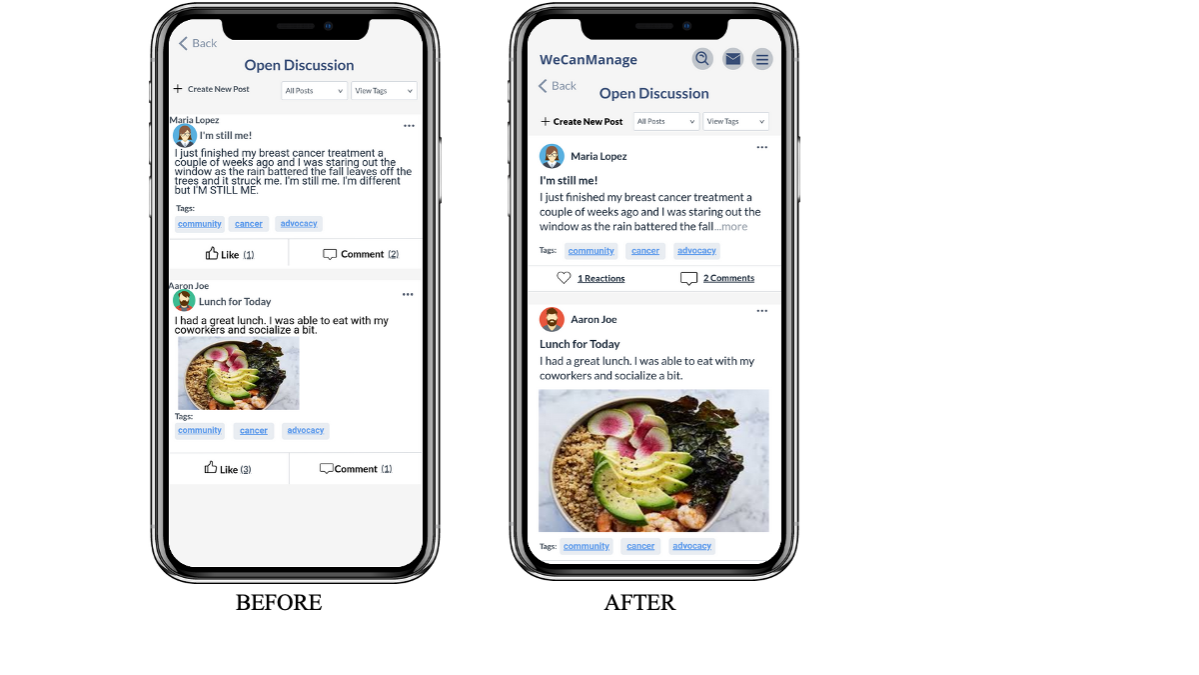
Modifications made to the Open Discussion design before and after usability testing .

On the basis of the findings from usability testing, we made several modifications to the prototype. To enhance usability, we increased the sizes of navigation icons, the eye icon, arrows within cards, and the top navigation bar. Throughout the application, we enlarged or bolded fonts for easier reading, including the “create new post” button in the Community section. We redesigned the layout of the Community Forum, increasing text and margins to achieve a cleaner and more concise design. Additionally, we revamped the subscribe button to reduce confusion (see Figures 7 and 8). To improve accessibility, we enlarged the C2P filter. Finally, we added a convenient way to return to the help guide by including it in the hamburger menu icon on the main page. These changes aim to enhance user experience and address the identified issues during usability testing.

## Discussion

### Principal Findings

Cancer and its treatments can lead to long-term disabilities, significantly impacting a survivor’s overall quality of life [10]. Unfortunately, postcancer treatment resources are often limited, further exacerbating the challenges faced by survivors [49,50]. To address this, we developed a high-fidelity prototype for an mHealth app called WeCanManage, aimed at empowering cancer survivors with disabilities to effectively self-manage the long-term effects of cancer treatment. Through conducting the heuristic evaluation, valuable improvements were made, including the incorporation of a helpful guide and the enhancement of accessibility formatting options, ultimately enhancing the overall user experience of the app.

In usability testing, we engaged cancer survivors with disabilities, using multiple methods such as task completion, think aloud strategies, SUS, perceived task satisfaction, and open-ended questions. These methods have been extensively used to evaluate various applications, with the SUS being one of the commonly used questionnaires [21]. The results of usability testing were overwhelmingly positive, with cancer survivors expressing appreciation for the app’s content, features, and design. The prototype achieved an impressive SUS score of 81, ranking it in the top 10% of scores and earning an A grade. Moreover, participants reported high satisfaction levels and efficiency, with average scores of 6.2 and 6.1 (out of 7), respectively. Conducting usability testing enabled us to thoroughly assess the app’s overall effectiveness, efficiency, satisfaction, and usability. We were able to identify areas for improvement, particularly in terms of accessibility. The insights gained from this testing process have allowed us to refine and enhance the app, ensuring a positive user experience for cancer survivors with disabilities.

In a study by Fuller-Tyszkiewicz et al [24], end users rated an mHealth prototype higher in usability and reported a more positive experience than clinical experts. Interestingly, users did not share the same concerns about the amount and layout of content presented as the experts had anticipated [24]. This discrepancy underscores the significance of testing potential users to tailor the app to their specific needs and preferences. While expert opinions (whether clinical or in design) are valuable, evaluating an app on actual users is ideal.

### Implications for Designers and Researchers

One of our primary findings is the importance of accessibility when designing applications for cancer survivors. Our app was specifically designed for cancer survivors with disabilities, and as such, we incorporated customized options to switch the learning style. Users could choose between clicking through content cards and accessing audio or text-only views. This flexibility proved to be helpful, particularly for participants with cognitive issues like “chemo brain,” who found it easier to navigate the audio versions of the course sessions. However, during testing, we identified other accessibility concerns related to font sizes and icons. Some users found them too small to see, click on, and navigate effectively. Addressing these issues is essential to ensure an inclusive and user-friendly experience for all app users.

The importance of having a help feature was revealed during heuristic evaluation, and through usability testing, we learned that users expressed a desire for a convenient way to return to the help guide. In response to this feedback, we have now incorporated the option to access the help guide directly from our main menu.

One comment expressed by many of our participants was how lonely the experience of a cancer survivor is. Consistent with findings from other studies that highlight the significance of social features in mHealth apps [51], participants expressed their appreciation for the Community Forum and C2P sections. These features provide a valuable opportunity for them to connect with others facing similar situations, fostering a sense of community and support. Additionally, participants reported that reading the content in the course sessions made them realize that their experiences were shared by others, helping them feel less isolated and reassured that they were not alone in their journey. When asked what they liked about the app, one participant wrote the following: “The information, reliable and trustworthy, … and the realization that I am not alone.”

### Limitations

Our aim was to achieve a minimum of 12 participants for usability testing, as SUS results are ideally derived from 12 or more participants [52,53]. However, we encountered challenges in recruitment because of technical difficulties, such as some participants lacking access to a laptop or facing issues with Zoom and screen sharing, leading to incomplete usability testing. Additionally, recruitment was hindered by our specific inclusion criteria, which focused on individuals who identified as having a disability. These challenges impacted our ability to reach the desired number of participants for the usability testing phase. Nevertheless, it is worth noting that according to Nielsen [54], 5 participants are typically adequate for identifying usability problems. Thus, we can reasonably infer that our processes have successfully identified the majority of issues, providing a level of confidence in the validity of our findings despite the lower number of participants in the usability testing phase. Additionally, it is worth mentioning that several studies evaluating mHealth prototypes have used the SUS with fewer than 12 participants [29,31,37]. We encountered instances where some participants experienced lingering effects of cancer and its treatment, but they did not self-identify as having a disability, resulting in their exclusion from usability testing. This finding has important implications for the implementation and adoption of WeCanManage, ensuring that cancer survivors experiencing disabling aftereffects can fully benefit from the tool and appreciate its relevance and value in their daily lives and experiences.

Furthermore, as this was a prototype, not all features were fully implemented (eg, the ability to create a post on the forum or direct message a user was mimicked), which may have caused some participants to encounter difficulties in the Community section of the prototype. In addition, during usability testing, participants expressed concerns regarding text and icon sizes. It is important to note that the testing was conducted over Zoom using computers (not mobile devices), and the prototype’s size (matching that of a phone) might have posed challenges during interaction, which may not be representative of the real application’s experience. Finally, it is worth noting that the age of participants and their level of comfort with technology might have influenced their overall experience [55]. Nevertheless, because these individuals constitute our target user base, it remains essential for us to maintain the app’s usability and accessibility to meet their needs.

### Conclusions

When creating an mHealth app, it is crucial to evaluate it with the target users in mind, in our case, cancer survivors with disabilities. Usability testing allowed us to identify the design’s strengths and areas requiring improvement. The WeCanManage prototype achieved a SUS score of 81, placing it in the top 10% of scores. Our future work will involve feasibility testing of an implemented web-based mobile app of WeCanManage. This will enable us to further refine the application and ensure that it meets the needs and preferences of our target users, enhancing its overall usability and impact.

## Abbreviations

C2P: Connect to Peers
mHealth: mobile health
SUS: System Usability Scale
